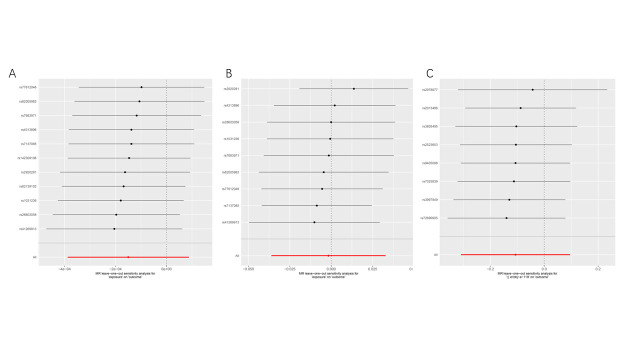# The relationship between periodontal disease and gastric cancer: A bidirectional Mendelian randomization study

**DOI:** 10.1097/MD.0000000000038490

**Published:** 2024-06-14

**Authors:** Ji-Chang Fan, Jin-Heng Gan, Hao Lu

**Affiliations:** a Department of Gastrointestinal Surgery, The Second Affiliated Hospital of Nanchang University, Nanchang, Jiangxi, China; b Jiangxi Province Key Laboratory of Molecular Medicine, The Second Affiliated Hospital of Nanchang University, Nanchang, Jiangxi, China.

**Keywords:** gastric cancer, mendelian randomization, periodontal

## Abstract

**Background:**

Previous observational studies have suggested a possible association between periodontal disease and gastric cancer (GC); however, a causal relationship has not yet been established. This study aimed to explore the causal relationship between the 2 through a 2-sample bidirectional Mendelian randomization (MR) study.

**Methods:**

Genome-wide association studies (GWAS) summary statistics were obtained from publicly available GWAS and relevant databases. Two-sample bidirectional MR analysis was conducted to investigate the causal relationship between periodontal disease and GC using the inverse-variance weighted (IVW) method selected as the primary analytical approach. Cochran Q test, MR-PRESSO, MR-pleiotropy, and leave-one-out analyses were performed to assess heterogeneity, pleiotropy, and sensitivity.

**Results:**

In European ancestry, IVW analysis revealed no causal relationship between periodontal disease and GC (OR = 1.873; 95% CI [4.788e-10, 7.323e + 09]; *P* = .956), or between loose teeth and GC (OR = 1.064; 95% CI [0.708, 1.598]; *P* = .765). In East Asian ancestry, there was no causal relationship between periodontitis and GC according to IVW (OR = 0.948; 95% CI [0.886, 1.015]; *P* = .126). Conversely, according to the results of the IVW analysis, there was no causal relationship between GC and periodontal disease, regardless of European or East Asian ancestry. Furthermore, there was no heterogeneity or pleiotropy in the causal relationships between these variables (all *P* > .05), suggesting a certain level of reliability in our results.

**Conclusion:**

Within the limitations of this MR study, we found no mutual causal relationship between periodontal disease and GC. This finding can prevent overtreatment by clinical physicians and alleviate the psychological burden on patients.

## 1. Introduction

According to a 2020 global cancer survey, gastric cancer (GC) accounts for 5.6% of the incidence and 7.7% of the mortality rates among common malignant tumors, posing a significant burden on human life.^[1]^ With the rapid advancement of sequencing technologies such as whole-genome sequencing and single-cell sequencing, the genomics of GC is gradually being unraveled, leading to a new era in molecular research.^[2–4]^ However, owing to its apparent heterogeneity, the etiology of GC requires further in-depth investigation. Current domestic and international research has found that *Helicobacter pylori* infection is a major risk factor for non-cardiac GC.^[5,6]^ Other contributing factors to GC include alcohol consumption, excessive intake of salt-preserved foods, and insufficient dietary fiber, with obesity and reflux being high-risk factors for cardiac cancer.^[7]^ Although GC exhibits a scattered distribution globally, approximately 10% of GC cases are closely associated with genetic factors.^[8,9]^ For instance, CDH1 mutations have been confirmed to be associated with hereditary diffuse GC.^[10]^ According to the latest theory proposed by Douglas Hanahan, microbial polymorphism is a characteristic of cancer.^[11]^ As the second largest microbial group after the gut, oral microbiota imbalance is often associated with the occurrence of diseases, such as GC, colorectal cancer, esophageal cancer, and other digestive tract tumors.^[12–14]^ This may be related to changes in microbial homeostasis and the release of systemic inflammatory mediators, ultimately leading to the occurrence and progression of tumors. In contrast, observational studies suggest that the risk of gastric adenocarcinoma increases by approximately 53% when periodontal disease is present.^[15]^ An observational study by Sun et al^[16]^ suggested an association between chronic periodontitis and precancerous lesions of GC, potentially mediated by increased expression of periodontal pathogenic bacteria, including *Tannerella forsythia, Treponema denticola*, and *Aggregatibacter actinomycetemcomitans*. Therefore, there is a theoretical correlation between periodontal disease and GC in terms of biology. Moreover, previous research on the relationship between periodontal disease and gastrointestinal malignancies has mainly focused on colorectal cancer, whereas studies on GC are limited. Thus, whether there is a direct causal relationship between periodontal disease and GC requires further investigation.

As one of the most common oral diseases, periodontal disease has become a global public health problem, with the cumulative number of people affected exceeding 10% to 15% of the total population.^[17]^ In the United States, the prevalence of periodontal disease among adults aged ≥ 30 years is 46%.^[18]^ Surprisingly, similar high prevalence rates have been observed in European populations,^[19]^ with a staggering 76% of individuals aged 65 to 74 in Germany affected by periodontal disease.^[20]^ Observational studies have found that the complexity of the oral microbiota in patients with GC is higher, which may be related to the lowered immunity of patients with cancer. When immunity is lowered, the oral cavity provides favorable conditions for bacterial reproduction. Additionally, compared with the control group, some pathogenic bacteria in the oral cavity of cancer patients increased by more than 2 times.^[21]^ However, observational studies are susceptible to confounding factors such as dietary habits, age, and environment. To date, no Mendelian randomization (MR) studies have utilized a bidirectional approach to investigate the causal relationship between GC and periodontal disease. Therefore, it is particularly important to explore the causal relationship between GC and periodontal diseases.

Currently, randomized controlled trials (RCT) are the gold standard for examining the relationship between exposure conditions and outcomes. However, certain types of RCT face challenges, such as lengthy experimental durations and difficulty in implementation, making them less feasible. In contrast, MR utilizes genetic variations as instrumental variables to study the causal relationships between exposure factors and outcome variables. This method effectively complements research that RCT have difficulty achieving.^[22]^ MR studies follow the random allocation principle of alleles, enabling them to effectively mitigate the influence of confounding factors between exposure and outcome variables. Since genetic mutations often occur before disease traits, using single nucleotide polymorphisms (SNPs) as instrumental variables can effectively avoid reverse causality.^[23]^ Therefore, MR studies can provide conclusive results regarding whether a causal relationship exists between exposure and outcome.

In this study, we propose the use of a bidirectional MR approach with a 2-sample analysis to explore the causal relationship between periodontal disease and GC, offering new insights into the relationship between gastric malignant tumors and periodontal disease.

## 2. Materials and methods

### 2.1. MR experimental design

**Figure 1.**
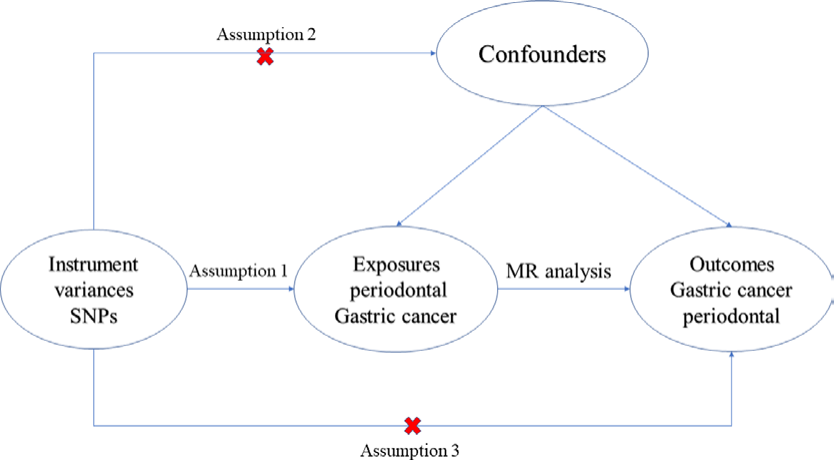
Schematic diagram of specific experimental design for MR analysis. MR = Mendelian randomization.

According to the steps of MR analysis, we first obtained relevant genetic variants as instrumental variables (IV), where SNP are commonly used as instrumental variables in MR analysis.^[24]^ In addition, the selection of IV must satisfy 3 assumptions: strong association between IVs and exposure conditions; No association between IVs and other known confounding factors. There was no association between the IVs and the outcome variable, and the IVs only affected the outcome variable through the exposure factor.^[25]^ A schematic diagram of the experimental design is shown in Figure 1.

### 2.2. Data source

The gingivitis and periodontal genome-wide association studies (GWAS) summary statistics for European ancestry were obtained from the UK Biobank database (http://www.nealelab.is/uk-biobank). The loose teeth summary statistics for the European ancestry and periodontitis statistics for the East Asian ancestry were sourced from the latest meta-analysis.^[26]^ In this study, periodontitis statistics were provided by the Gene-Lifestyle Interactions in Dental Endpoints (GLIDE) consortium, and the status of periodontal disease was assessed based on the Centers for Disease Control and Prevention/American Academy of Periodontology (CDC/AAP) criteria.^[27]^ GC GWAS summary statistics for European ancestry were obtained from the FinnGen database (R9 version) (https://www.finngen.fi/). GC summary statistics for East Asian ancestry were obtained from the BBJ database accessed through the IEU database (https://gwas.mrcieu.ac.uk/). The GWAS datasets for analysis were obtained from a public platform; therefore, no ethical approval was required.

### 2.3. Genetic instrument selection

Using the “TwoSampleMR” package in R Studio (version 4.2.3), we read the downloaded GWAS summary statistics. To identify SNPs strongly associated with the exposure factor, we initially set the threshold for association at *P* < 5e-08. However, due to the limited data available, we adjusted the association threshold to *P* < 5e-06. Subsequently, we used the “Clump” function to remove linkage disequilibrium (LD), setting the independence criteria as r^2^ < 0.001 and kb > 10000 to account for LD.^[24]^ We calculated the statistical strength using *F* = beta^2^/se^2^ and excluded SNPs with *F* < 10.^[28]^ Each SNP was filtered for confounding factors using the Phenoscanner website (http://www.phenoscanner.medschl.cam.ac.uk/), with a filtering criterion of *P* < 5e-08 for confounders such as smoking,^[29]^ and alcohol consumption^[30]^ that may influence the outcome. Conversely, we used the same methods and parameters to select SNPs that were strongly associated with GC as exposure factors for MR analysis.

### 2.4. Statistical analysis

We selected inverse-variance weighted (IVW),^[31]^ weight median,^[32]^ weight mode, mr-egger^[33]^ and maximum-likelihood^[34]^ as the analysis methods. While each method has its focus, the IVW method is particularly important, as it can indicate the presence of a causal relationship between the exposure factor and the outcome variable. Finally, we used Cochran Q, MR-pleiotropy, MR-PRESSO,^[35]^ and leave-one-out tests to assess the sensitivity and accuracy of the results.^[24]^

## 3. Results

### 3.1. The causal relationship between periodontal disease and GC

First, in European ancestry, our dataset on gingivitis and periodontal disease from the UK Biobank database included 458 cases and 360,736 controls (Supplementary Table 1, http://links.lww.com/MD/M809). GC in the FinnGen was the outcome, which included 1307 cases and 287,137 controls (Supplementary Table 1, http://links.lww.com/MD/M809). After allele harmonization, 32 SNPs were included in the MR analysis, and all SNPs had *F* > 10, indicating no weakness in our choice of instrumental variables (Supplementary Table 2, http://links.lww.com/MD/M810). However, the results from the IVW analysis indicated no causal relationship between periodontal disease and GC (OR = 1.873; 95% CI [4.788e-10, 7.323e + 09]; *P* = .956). At the same time, we obtained GWAS statistics for 18,979 cases and 442,052 controls from GLIDE consortium. When loose teeth were used as exposure factors, 12 SNPs were obtained for MR analysis (Supplementary Table 3, http://links.lww.com/MD/M811). The results of the IVW analysis were (OR = 1.064; 95% CI [0.708, 1.598]; *P* = .765).

**Figure 2.**
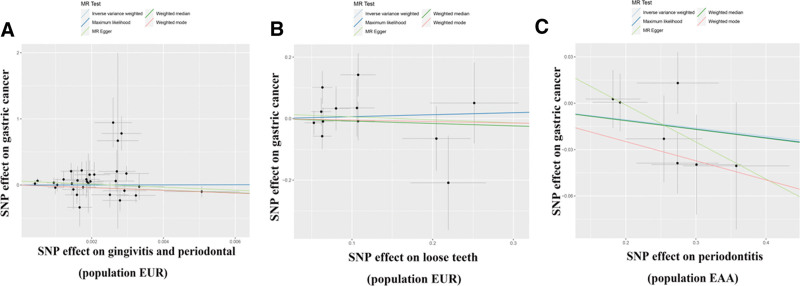
Scatter plot of causal relationship between periodontal disease and gastric cancer analyzed by different MR methods. (A) Causal relationship between gingivitis periodontal and gastric cancer in a European ancestry. (B) Causal relationship between loose teeth and gastric cancer in a European ancestry. (C) Causal relationship between periodontitis and gastric cancer in an East Asian ancestry. MR = Mendelian randomization.

To mitigate the impact of racial ancestry on our results. In East Asian ancestry, periodontitis will be considered as the exposure condition, which included 1680 cases and 15,607 controls. And GC in the BBJ dataset was the outcome, which included 6563 cases and 195,745 controls. We employed the same criteria to screen the data; as a result, we obtained 7 SNPs for MR analysis (Supplementary Table 4, http://links.lww.com/MD/M812). Interestingly, the IVW analysis results similarly supported the absence of a causal relationship between periodontal disease and GC (OR = 0.948; 95%CI [0.886,1.015]; *P* = .126). Moreover, alternative MR analysis methods such as maximum-likelihood, MR-Egger, weighted median, and weighted mode approaches consistently provided support for the absence of a direct causal relationship between periodontal diseases and GC (*P* > .05) (Table 1 and Fig. 2). Cochran Q, MR-PRESSO, and MR-pleiotropy results showed that all *P* values were >.05, indicating the reliability of our conclusions (Table 2 and Fig. S1, http://links.lww.com/MD/M805). We used the leave-one-out method to gradually eliminate each SNP and found that our results were not significantly affected (Fig. S2, http://links.lww.com/MD/M806). Therefore, our results suggest that there is no causal relationship between periodontal disease and GC.

**Table 1:** The causal relationship between periodontal disease and gastric cancer was analyzed by different MR methods.

Exposure	Outcome	Pop	SNPs	Method	OR	95%CI	*P* value
Gingivitis periodontal	Gastric cancer	EUR	32	IVW	1.873	4.788e-10, 7.323e + 09	.956
				Maximum-likelihood	1.915	2.776e-09, 1.321e + 09	.95
				MR-Egger	1.427e-10	8.622e-25, 2.362e + 04	.185
				Weighted median	3.129e-09	1.126e-22, 8.693e + 04	.215
				Weighted mode	1.687e-09	8.53e-22, 3.334e + 03	.172
Periodontitis	Gastric cancer	EAA	7	IVW	0.948	0.886, 1.105	.126
				Maximum-likelihood	0.946	0.881, 1.015	.121
				MR-Egger	0.787	0.563, 1.100	.220
				Weighted median	0.945	0.859, 1.040	.245
				Weighted mode	0.883	0.754, 1.035	.175
Loose teeth	Gastric cancer	EUR	12	IVW	1.064	0.708, 1.598	.765
				Maximum-likelihood	1.067	0.724, 1.572	.744
				MR-Egger	0.908	0.353, 2.335	.845
				Weighted median	0.924	0.539, 1.584	.775
				Weighted mode	0.953	0.456, 1.993	.901
Gastric cancer	Gingivitis periodontal	EUR	11	IVW	9.999e-01	9.996e-01, 1	.217
				Maximum-likelihood	9.998e-01	9.996e-01, 1	.212
				MR-Egger	1	9.996e-01, 1.001	.806
				Weighted median	9.999e-01	9.995e-01, 1	.453
				Weighted mode	1	9.996e-01, 1	.955
Gastric cancer	Periodontitis	EAA	8	IVW	0.899	0.734, 1.101	.302
				Maximum-likelihood	0.899	0.733, 1.101	.302
				MR-Egger	0.851	0.442, 1.638	.646
				Weighted median	0.872	0.677, 1.124	.291
				Weighted mode	0.852	0.664, 1.094	.250
Gastric cancer	Loose teeth	EUR	9	IVW	0.998	0.965, 1.033	.925
				Maximum-likelihood	0.998	0.968, 1.03	.914
				MR-Egger	1.022	0.944, 1.105	.613
				Weighted median	1.006	0.965, 1.05	.771
				Weighted mode	1.012	0.955, 1.072	.697

IVW = inverse-variance weighted, MR = Mendelian randomization.

**Table 2:** Heterogeneity and pleiotropy testing of mutual causality between periodontal disease and gastric cancer.

Exposure	Outcome	Pop	Heterogeneity		Mr-presso	Pleiotropy		
			Q	*P*	Global Test *P* val	Intercept	se	*P*
Gingivitis periodontal	Gastric cancer	EUR	38.769	.159	.155	0.059	0.032	.076
Periodontitis	Gastric cancer	EAA	5.1	.53	.493	0.047	0.042	.316
Loose teeth	Gastric cancer	EUR	12.62	.319	.34	0.016	0.042	.72
Gastric cancer	Gingivitis periodontal	EUR	7.342	.693	.705	-7.55e-05	8.08e-05	.374
Gastric cancer	Periodontitis	EAA	2.824	.901	.900	0.010	0..058	.868
Gastric cancer	Loose teeth	EUR	10.209	.251	.253	-0.008	0.012	.543

### 3.2. The causal relationship between GC and periodontal disease

In contrast, to investigate the causal relationship between GC and periodontal diseases, we considered GC as the exposure factor and gingivitis, periodontal, and loose teeth diseases as the outcome in European ancestry. When gingivitis and periodontal diseases were used as outcomes, after allele harmonization, we obtained 11 SNPs for the MR analysis (Supplementary Table 5, http://links.lww.com/MD/M813). The IVW analysis showed no causal relationship between GC and periodontal disease (OR = 0.999; 95% CI [0.9996, 1]; *P* = .217). Next, when loose teeth were the outcome, we obtained 9 SNPs for MR analysis (Supplementary Table 6, http://links.lww.com/MD/M814). The IVW analysis also showed no causal relationship between loose teeth and GC (OR = 0.998; 95% CI [0.965, 1.033]; *P* = .925).

**Figure 3.**
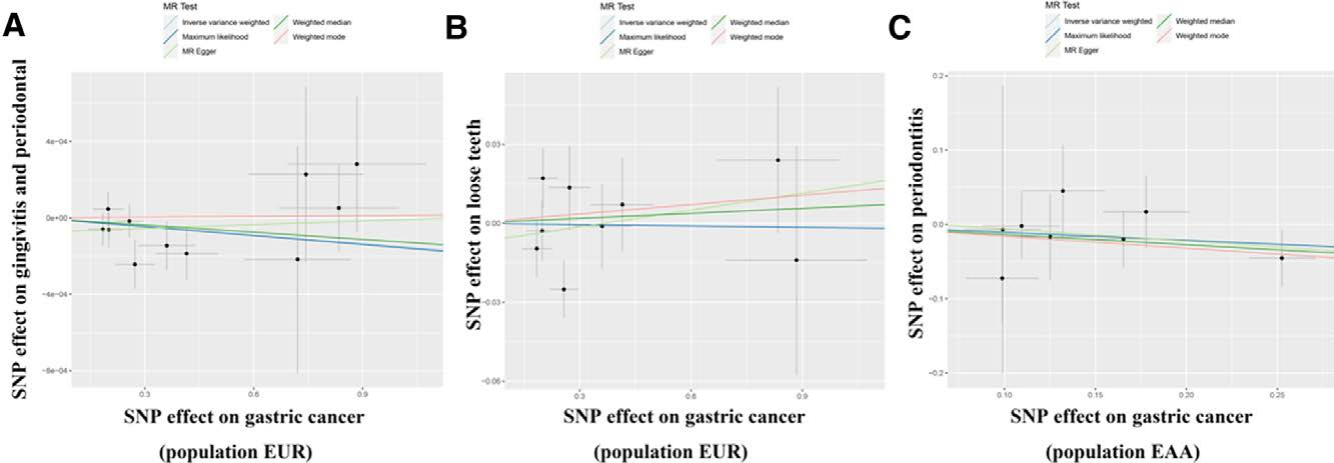
Scatter plot of causal relationship between gastric cancer and periodontal disease analyzed by different MR methods. (A) Causal relationship between gastric cancer and gingivitis periodontal in a European ancestry. (B) Causal relationship between gastric cancer and loose teeth in a European ancestry. (C) Causal relationship between gastric cancer and periodontitis in an East Asian ancestry. MR = Mendelian randomization.

To avoid the influence of race on our results, we extracted exposure factors of East Asian ancestry according to the same screening conditions. We obtained 8 SNPs for MR analysis (Supplementary Table 7, http://links.lww.com/MD/M815). The results showed IVW (OR = 0.899; 95% CI [0.734, 1.101]; *P* = .303). These results suggest that there was no causal relationship between GC and periodontal disease (Table 1 and Fig. 3). At the same time, we performed sensitivity and heterogeneity tests for the above 3 causal relationships, and all *P* values were >.05 (Table 2). These findings indicate that our MR analysis did not exhibit heterogeneity or pleiotropy, and the leave-one-out sensitivity analysis also confirmed the accuracy of our results (Fig. S3-S4, http://links.lww.com/MD/M807, http://links.lww.com/MD/M808). Therefore, we believe that there is no causal relationship between GC and periodontal diseases.

## 4. Discussion

In this study, we investigated the bidirectional causal relationship between periodontal disease and GC at the genetic susceptibility level using MR analysis for the first time. Interestingly, our findings demonstrated that there is no causal relationship between periodontal disease and GC in European and Asian populations. Conversely, there was no causal relationship between GC and periodontal disease. Therefore, as a clinician, especially a dental practitioner, since there is no causal relationship between periodontal disease and GC, it is possible to reduce unnecessary medical interventions and provide patients with a reasonable explanation to alleviate their psychological burden.

Previous studies have suggested an increased risk of certain malignancies and mortality associated with periodontal diseases. For example, Verma et al^[36]^ conducted a meta-analysis that showed that chronic periodontitis can increase the incidence of lung cancer. Beger-Luedde et al^[37]^ in a retrospective study of 19,782 individuals in the UK, Beger-Luedde et al found a significantly higher risk of various types of cancer (including breast cancer, lymphoid system cancer, and digestive tract cancers) in patients with chronic gingivitis, particularly prostate cancer. However, the specific mechanisms underlying the association between periodontal diseases and tumors remain unclear, and some researchers have suggested that it may be a combination of systemic inflammatory responses, immune stress, and microbial dysbiosis. First, chronic periodontitis can lead to the release of inflammatory mediators into the bloodstream, thereby triggering a systemic inflammatory response. Inflammatory factors such as IL-6, IL-1, and Tregs have been implicated,^[38]^ and studies have shown an association of over 15% between chronic inflammation and cancer.^[39]^ Second, periodontitis can induce immune system stress^[40]^; for example, it can cause T-cell dysfunction^[41]^ and immune dysregulation is known to be a risk factor for cancer. Lastly, microbial dysbiosis is also considered to be one of the factors linking periodontal diseases to tumor development. Researchers have found that oral bacteria can migrate to the colon through the bloodstream or oral-gut axis, leading to pathogenesis.^[42]^ However, our study found no causal relationship between periodontal diseases and GC, which is partially consistent with the prospective study by Ndegwa et al.^[43]^ Their research showed that higher levels of dental plaque do not significantly increase the risk of GC, and that the relationship between periodontal diseases and GC is largely confounded by age. *Porphyromonas gingivalis*, one of the major pathogenic bacteria in periodontal diseases, can survive in the oral cavity and contribute to the development of malignant tumors. However, studies have found that the activity of this bacterium is significantly inhibited by gastric acid and bile, and it cannot survive at pH levels below 2.5, whereas gastric acid (pH 0.9–1.8) is much lower than this threshold,^[44]^ This may provide a reasonable explanation for the lack of a causal relationship between *P gingivalis* and GC.

On the other hand, our study found no causal relationship between GC and periodontal diseases, which is inconsistent with previous observational studies suggesting an increased risk of oral complications in cancer patients.^[45]^ Although there is a general understanding that the occurrence of periodontal disease is higher in some cancer patients than in the general population, we found no causal relationship between GC and periodontal disease, which may be attributed to other confounding factors. For example, in GC, chemotherapy or immunotherapy can cause oral mucosal damage,^[46]^ which provides favorable conditions for the colonization of pathogenic bacteria. Additionally, Nicolae et al found that oral microbiota diversity increases in patients with GC, with Fusobacterium nucleatum and *T forsythia* being the most prominent bacteria, and these 2 bacteria may contribute to the occurrence of periodontal diseases.^[14,47]^ Furthermore, a study reported a significant decrease in tooth brushing frequency among hospitalized patients with GC, especially those who underwent surgery or had a longer hospital stay, leading to the formation of dental plaque and subsequent periodontal diseases.^[48]^ Therefore, the increased risk of periodontal disease in patients with GC observed in observational studies can be explained by confounding factors, such as age and chemotherapy-induced syndrome. While our study suggests that GC does not cause periodontal disease, it is still crucial for clinical physicians, particularly those caring for postoperative patients, to pay special attention to oral hygiene to minimize the risk of periodontal disease.

Our study has some limitations. First, the study population consisted of individuals diagnosed with periodontal disease. This may not represent the true prevalence of the condition, as in economically disadvantaged regions, a large portion of the population may have less awareness of periodontal disease and may not seek dental care. Second, owing to the inclusion of a limited number of SNPs, we set relatively stringent criteria for the association, which may affect the accuracy of the results.

## 5. Conclusions

In this study, using MR analysis, we found no causal relationship between periodontal disease and GC, and vice versa. However, given the complex relationship between periodontal diseases and GC, further experimental or mediation MR analyses are needed to further explore their association and obtain more credible evidence to support these findings.

## Author contributions

**Conceptualization:** Ji-Chang Fan.

**Data curation:** Jin-Heng Gan.

**Software:** Jin-Heng Gan.

**Supervision:** Hao Lu.

**Writing – original draft:** Ji-Chang Fan.

**Writing – review & editing:** Hao Lu.

## Supplementary Material















**Figure:**
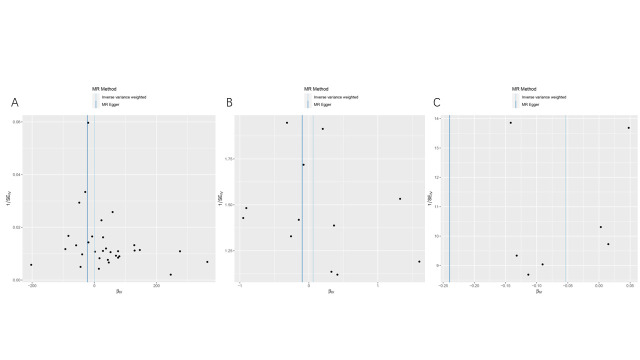


**Figure:**
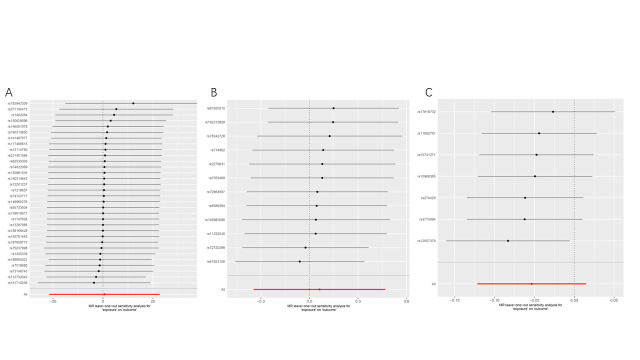


**Figure:**
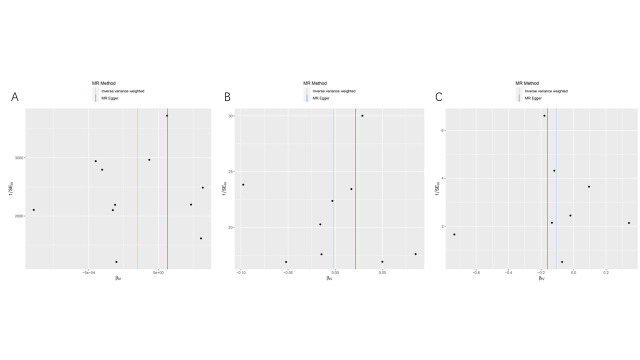


**Figure:**