# Supplementary material for: The relationship between periodontal disease and gastric cancer: A bidirectional Mendelian randomization study
Source: Medicine (Baltimore). 2024 Jun 14;103(24):e38490. doi: 10.1097/MD.0000000000038490 (PMC11175918; doi:10.1097/MD.0000000000038490)
Supplement: Supplementary file 1 [file medi-103-e38490-s001.docx]

**Supplementary Table 1 Data source and sample size of GWAS summary statistics**

| **Traits** | **Consortium** | **Sample size** | **N case** | **N control** | **Population** |
| --- | --- | --- | --- | --- | --- |
| Gingivitis Periodontal | UKB | 361194 | 458 | 360736 | European |
| Periodontitis | GLIDE | 17287 | 1680 | 15607 | East Asia |
| Loose teeth | UKB/GLIDE | 461,031 | 18,979 | 442,052 | European |
| Gastric cancer | BBJ | 202308 | 6,563 | 195,745 | East Asia |
| Gastric cancer | FinnGen | 288444 | 1307 | 287137 | European |
