# Supplementary material for: The relationship between periodontal disease and gastric cancer: A bidirectional Mendelian randomization study
Source: Medicine (Baltimore). 2024 Jun 14;103(24):e38490. doi: 10.1097/MD.0000000000038490 (PMC11175918; doi:10.1097/MD.0000000000038490)
Supplement: Supplementary file 2 [file medi-103-e38490-s002.docx]

**Supplementary Table 2** **Characteristics of genetic variants associated with gingivitis periodontal and their effect on GC in European ancestry**

|  |  | **Gingivitis and periodontal(exposure)** | | | **Gastric cancer(outcome)** | | |  |
| --- | --- | --- | --- | --- | --- | --- | --- | --- |
| **SNP** | **Effect allele** | **beta** | **se** | **pval** | **beta** | **se** | **pval** | ***F*** |
| rs112732042 | A | 0.0028323 | 0.000551 | 2.80E-07 | 0.780184 | 0.25935 | 0.00262773 | 26.38655 |
| rs117468815 | T | 0.0017603 | 0.00037 | 2.00E-06 | -0.0334579 | 0.123211 | 0.785968 | 22.59805 |
| rs12201237 | A | 0.0014488 | 0.000309 | 2.81E-06 | 0.0239607 | 0.175073 | 0.891141 | 21.94505 |
| rs13114760 | T | 0.0009875 | 0.000213 | 3.39E-06 | -0.0386487 | 0.101363 | 0.702987 | 21.58141 |
| rs13287986 | T | 0.0012189 | 0.000261 | 3.09E-06 | 0.0846842 | 0.131885 | 0.520805 | 21.76366 |
| rs139910917 | A | 0.0027021 | 0.000521 | 2.19E-07 | 0.206185 | 0.318234 | 0.517047 | 26.85906 |
| rs1402264 | G | -0.002914 | 0.000611 | 1.86E-06 | 0.0854498 | 0.087181 | 0.327014 | 22.73479 |
| rs140314942 | T | 0.0027284 | 0.000582 | 2.74E-06 | 0.670219 | 1.32824 | 0.613845 | 21.9937 |
| rs1405338 | G | 0.0004925 | 9.83E-05 | 5.48E-07 | 0.0637492 | 0.043986 | 0.147255 | 25.09837 |
| rs141487877 | T | 0.0016653 | 0.000344 | 1.31E-06 | -0.337996 | 0.287698 | 0.240063 | 23.4051 |
| rs141710256 | A | 0.0029678 | 0.000629 | 2.34E-06 | 0.174414 | 0.115045 | 0.129507 | 22.29697 |
| rs145701443 | G | 0.0019665 | 0.00038 | 2.31E-07 | 0.0551228 | 0.121276 | 0.649452 | 26.7587 |
| rs146051978 | T | 0.0015892 | 0.000328 | 1.31E-06 | -0.148131 | 0.135237 | 0.273365 | 23.40952 |
| rs148960276 | T | 0.0018711 | 0.000379 | 8.12E-07 | 0.0517992 | 0.169371 | 0.759733 | 24.33033 |
| rs150629599 | C | 0.0027893 | 0.000484 | 8.19E-09 | -0.232546 | 0.166514 | 0.162547 | 33.23048 |
| rs150881535 | G | 0.0019077 | 0.000412 | 3.57E-06 | 0.0278432 | 0.445377 | 0.950152 | 21.4845 |
| rs1747626 | A | 0.0004201 | 8.62E-05 | 1.08E-06 | 0.0218699 | 0.03967 | 0.581428 | 23.75545 |
| rs183943359 | T | 0.0050669 | 0.001047 | 1.31E-06 | -0.100057 | 0.084903 | 0.238605 | 23.40591 |
| rs187659773 | T | 0.0020727 | 0.000449 | 3.95E-06 | 0.158149 | 0.18942 | 0.403769 | 21.28929 |
| rs189168428 | C | 0.0019426 | 0.000417 | 3.25E-06 | 0.15627 | 0.215692 | 0.468756 | 21.6651 |
| rs189955301 | C | 0.0025989 | 0.000525 | 7.24E-07 | 0.944501 | 0.377499 | 0.0123495 | 24.55066 |
| rs190313850 | G | 0.0025088 | 0.000523 | 1.58E-06 | -0.146836 | 0.190415 | 0.440626 | 23.05291 |
| rs371192413 | T | 0.0032329 | 0.000542 | 2.39E-09 | -0.156896 | 0.110111 | 0.15419 | 35.63199 |
| rs531401566 | G | 0.0033239 | 0.000699 | 2.00E-06 | -0.0234063 | 0.200963 | 0.90728 | 22.59919 |
| rs55733934 | A | 0.0009446 | 0.000199 | 2.06E-06 | 0.0354756 | 0.078888 | 0.652929 | 22.54214 |
| rs62530300 | C | 0.001491 | 0.000316 | 2.46E-06 | -0.0661632 | 0.298641 | 0.824666 | 22.19598 |
| rs7219657 | A | 0.0018534 | 0.000371 | 6.00E-07 | 0.0860315 | 0.278594 | 0.757469 | 24.91443 |
| rs73148743 | T | 0.001724 | 0.000362 | 1.91E-06 | 0.221459 | 0.130356 | 0.0893429 | 22.68297 |
| rs74622069 | G | 0.0010418 | 0.000228 | 4.94E-06 | 0.00190554 | 0.096846 | 0.984302 | 20.85902 |
| rs74743717 | A | 0.0016064 | 0.000343 | 2.79E-06 | 0.0697702 | 0.209704 | 0.739355 | 21.9582 |
| rs7518680 | T | 0.0014274 | 0.000286 | 6.18E-07 | 0.208776 | 0.125242 | 0.0955191 | 24.85613 |
| rs75237998 | A | 0.0026702 | 0.000515 | 2.19E-07 | 0.0610802 | 0.117802 | 0.604109 | 26.86131 |
