# Supplementary material for: The relationship between periodontal disease and gastric cancer: A bidirectional Mendelian randomization study
Source: Medicine (Baltimore). 2024 Jun 14;103(24):e38490. doi: 10.1097/MD.0000000000038490 (PMC11175918; doi:10.1097/MD.0000000000038490)
Supplement: Supplementary file 3 [file medi-103-e38490-s003.docx]

**Supplementary Table 3** **Characteristics of genetic variants associated with loose teeth and their effect on GC in European ancestry**

|  |  | **Loose teeth(exposure)** | | | **Gastric cancer(outcome)** | | | |  |
| --- | --- | --- | --- | --- | --- | --- | --- | --- | --- |
| **SNP** | **Effect allele** | **beta** | **se** | **pval** | | **beta** | **se** | **pval** | ***F*** |
| rs11220245 | A | -0.062 | 0.0124 | 5.90E-07 | | -0.02263 | 0.044692 | 0.612654 | 25 |
| rs145982086 | A | -0.2515 | 0.0549 | 4.66E-06 | | -0.05077 | 0.131431 | 0.699309 | 20.98608 |
| rs192315959 | A | 0.2194 | 0.047 | 3.09E-06 | | -0.20873 | 0.153623 | 0.174244 | 21.79102 |
| rs2278831 | A | 0.1076 | 0.0207 | 2.13E-07 | | -0.00825 | 0.062625 | 0.895144 | 27.01991 |
| rs3763469 | T | 0.0641 | 0.0131 | 9.64E-07 | | -0.00936 | 0.045191 | 0.835994 | 23.94272 |
| rs61823158 | A | 0.1077 | 0.0219 | 8.30E-07 | | 0.142499 | 0.070266 | 0.042562 | 24.18484 |
| rs61920210 | T | 0.0631 | 0.0115 | 4.23E-08 | | -0.05722 | 0.04257 | 0.178939 | 30.10669 |
| rs6586364 | T | 0.0803 | 0.0174 | 3.91E-06 | | 0.033232 | 0.071707 | 0.643055 | 21.29769 |
| rs714962 | A | -0.0528 | 0.0115 | 4.54E-06 | | 0.013653 | 0.039742 | 0.731198 | 21.08008 |
| rs72664597 | A | -0.1062 | 0.0217 | 1.00E-06 | | -0.03475 | 0.093523 | 0.710194 | 23.95133 |
| rs72720396 | A | 0.0634 | 0.0127 | 6.07E-07 | | 0.101569 | 0.053245 | 0.056446 | 24.92132 |
| rs78242728 | A | -0.2046 | 0.0424 | 1.42E-06 | | 0.064899 | 0.105097 | 0.536895 | 23.28518 |
