# Supplementary material for: The relationship between periodontal disease and gastric cancer: A bidirectional Mendelian randomization study
Source: Medicine (Baltimore). 2024 Jun 14;103(24):e38490. doi: 10.1097/MD.0000000000038490 (PMC11175918; doi:10.1097/MD.0000000000038490)
Supplement: Supplementary file 4 [file medi-103-e38490-s004.docx]

**Supplementary Table 4 Characteristics of genetic variants associated with periodontitis and their effect on GC in East Asian ancestry**

|  |  | **Periodontitis(exposure)** | | | **Gastric cancer(outcome)** | | |  |
| --- | --- | --- | --- | --- | --- | --- | --- | --- |
| **SNP** | **Effect allele** | **beta** | **se** | **pval** | **beta** | **se** | **pval** | ***F*** |
| rs10741271 | A | 0.3576 | 0.0758 | 2.35E-06 | -0.04053 | 0.041155 | 0.324748 | 22.25649 |
| rs10968385 | T | -0.2543 | 0.055 | 3.80E-06 | 0.023006 | 0.028138 | 0.41358 | 21.37801 |
| rs11982781 | T | -0.3008 | 0.0643 | 2.91E-06 | 0.039709 | 0.032216 | 0.217736 | 21.88435 |
| rs12507379 | A | -0.2741 | 0.0586 | 2.92E-06 | -0.01307 | 0.020029 | 0.513934 | 21.87877 |
| rs17818702 | A | 0.2737 | 0.0586 | 2.94E-06 | -0.03875 | 0.01975 | 0.049763 | 21.81496 |
| rs274428 | T | -0.1918 | 0.0391 | 9.33E-07 | -0.00056 | 0.018606 | 0.97578 | 24.06266 |
| rs3773494 | A | -0.1816 | 0.039 | 3.21E-06 | -0.00272 | 0.01867 | 0.88396 | 21.68216 |
