# Supplementary material for: The relationship between periodontal disease and gastric cancer: A bidirectional Mendelian randomization study
Source: Medicine (Baltimore). 2024 Jun 14;103(24):e38490. doi: 10.1097/MD.0000000000038490 (PMC11175918; doi:10.1097/MD.0000000000038490)
Supplement: Supplementary file 5 [file medi-103-e38490-s007.docx]

**Supplementary Table 5** **Characteristics of genetic variants associated with gastric cancer and their effect on gingivitis periodontal in European ancestry**

|  |  | **Gastric cancer（exposure）** | | | **Gingivitis and periodontal (outcome)** | | |  |
| --- | --- | --- | --- | --- | --- | --- | --- | --- |
| **SNP** | **Effect allele** | **beta** | **se** | **pval** | **beta** | **se** | **pval** | ***F*** |
| rs1031236 | G | 0.198819 | 0.042557 | 2.99E-06 | 4.67E-05 | 9.05E-05 | 0.606224 | 21.82567 |
| rs142309106 | C | 0.720788 | 0.145714 | 7.55E-07 | -0.000216802 | 0.000595 | 0.71577 | 24.46879 |
| rs28603058 | T | 0.884102 | 0.191161 | 3.75E-06 | 0.000282075 | 0.000355 | 0.427376 | 21.38977 |
| rs2920281 | T | 0.257449 | 0.038837 | 3.38E-11 | -1.67E-05 | 8.68E-05 | 0.847153 | 43.94404 |
| rs41269913 | T | -0.83446 | 0.164768 | 4.10E-07 | -5.24E-05 | 0.000224 | 0.815151 | 25.64882 |
| rs4313896 | G | -0.18431 | 0.039954 | 3.97E-06 | 6.02E-05 | 8.77E-05 | 0.492637 | 21.28051 |
| rs62005983 | A | -0.41498 | 0.085365 | 1.17E-06 | 0.000186531 | 0.000141 | 0.185774 | 23.63156 |
| rs62139102 | T | -0.74384 | 0.157957 | 2.49E-06 | -0.000228065 | 0.00046 | 0.620331 | 22.17584 |
| rs7137085 | A | 0.200796 | 0.040171 | 5.78E-07 | -6.24E-05 | 9.16E-05 | 0.495589 | 24.98507 |
| rs7683971 | C | -0.36041 | 0.078816 | 4.81E-06 | 0.000145372 | 0.000129 | 0.259439 | 20.91051 |
| rs77612046 | T | -0.27254 | 0.054902 | 6.90E-07 | 0.000243712 | 0.000129 | 0.059696 | 24.64193 |
