# Supplementary material for: The relationship between periodontal disease and gastric cancer: A bidirectional Mendelian randomization study
Source: Medicine (Baltimore). 2024 Jun 14;103(24):e38490. doi: 10.1097/MD.0000000000038490 (PMC11175918; doi:10.1097/MD.0000000000038490)
Supplement: Supplementary file 6 [file medi-103-e38490-s008.docx]

**Supplementary Table 6 Characteristics of genetic variants associated with gastric cancer and their effect on loose teeth in European ancestry**

|  |  | **Gastric cancer(exposure)** | | | **Loose teeth (outcome)** | | |  |
| --- | --- | --- | --- | --- | --- | --- | --- | --- |
| **SNP** | **Effect allele** | **beta** | **se** | **pval** | **beta** | **se** | **pval** | ***F*** |
| rs1031236 | G | 0.198819 | 0.042557 | 2.99E-06 | -0.0029 | 0.0113 | 0.7997 | 21.82567 |
| rs28603058 | T | 0.884102 | 0.191161 | 3.75E-06 | -0.014 | 0.0436 | 0.7483 | 21.38977 |
| rs2920281 | T | 0.257449 | 0.038837 | 3.38E-11 | -0.0251 | 0.0108 | 0.02033 | 43.94404 |
| rs41269913 | T | -0.83446 | 0.164768 | 4.10E-07 | -0.024 | 0.0278 | 0.3886 | 25.64882 |
| rs4313896 | G | -0.18431 | 0.039954 | 3.97E-06 | 0.0096 | 0.0109 | 0.381 | 21.28051 |
| rs62005983 | A | -0.41498 | 0.085365 | 1.17E-06 | -0.0071 | 0.0177 | 0.6884 | 23.63156 |
| rs7137085 | A | 0.200796 | 0.040171 | 5.78E-07 | 0.0171 | 0.0114 | 0.134 | 24.98507 |
| rs7683971 | C | -0.36041 | 0.078816 | 4.81E-06 | 0.0011 | 0.0161 | 0.9475 | 20.91051 |
| rs77612046 | T | -0.27254 | 0.054902 | 6.90E-07 | -0.0136 | 0.0161 | 0.3985 | 24.64193 |
