# Supplementary material for: The relationship between periodontal disease and gastric cancer: A bidirectional Mendelian randomization study
Source: Medicine (Baltimore). 2024 Jun 14;103(24):e38490. doi: 10.1097/MD.0000000000038490 (PMC11175918; doi:10.1097/MD.0000000000038490)
Supplement: Supplementary file 7 [file medi-103-e38490-s009.docx]

**Supplementary Table 7 Characteristics of genetic variants associated with gastric cancer and their effect on periodontitis in East Asian ancestry**

|  |  | **Gastric cancer(exposure)** | | | **Periodontitis (outcome)** | | |  |
| --- | --- | --- | --- | --- | --- | --- | --- | --- |
| **SNP** | **Effect allele** | **beta** | **se** | **pval** | **beta** | **se** | **pval** | ***F*** |
| rs2013486 | T | -0.09878 | 0.019943 | 7.30E-07 | 0.0725 | 0.0596 | 0.224 | 24.53357 |
| rs2523653 | G | 0.125161 | 0.02347 | 9.67E-08 | -0.0167 | 0.0583 | 0.7744 | 28.4386 |
| rs2978977 | A | 0.252482 | 0.018304 | 2.78E-43 | -0.0453 | 0.0382 | 0.2349 | 190.2673 |
| rs3805495 | T | -0.16541 | 0.018174 | 8.92E-20 | 0.0196 | 0.0383 | 0.6083 | 82.83491 |
| rs3997849 | T | 0.13212 | 0.023036 | 9.73E-09 | 0.0451 | 0.0618 | 0.4655 | 32.89533 |
| rs7025839 | A | 0.109417 | 0.021296 | 2.78E-07 | -0.0021 | 0.0447 | 0.9624 | 26.39716 |
| rs72690905 | T | -0.17785 | 0.023389 | 2.87E-14 | -0.0169 | 0.0487 | 0.7288 | 57.82332 |
| rs9405098 | A | 0.099089 | 0.020721 | 1.73E-06 | -0.007 | 0.1938 | 0.9712 | 22.86842 |
